# Sleeping in multicultural societies: The longitudinal interplay between adolescents’ sleep health and intercultural interactions

**DOI:** 10.1016/j.ijchp.2026.100680

**Published:** 2026-04-09

**Authors:** Maria Pagano, Valeria Bacaro, Elisabetta Crocetti

**Affiliations:** Department of Psychology “Renzo Canestrari”, University of Bologna, Viale Berti Pichat 5, Bologna (BO), Viale Europa 115, Cesena, FC 47521, Italy

**Keywords:** Sleep health, Adolescents, Longitudinal study, Intercultural interactions, Actigraphy

## Abstract

Adolescents’ sleep is intertwined with their well-being and daily life experiences. Sleep health is conceptualized as a multidimensional construct encompassing complementary components, including subjective dimensions (e.g., perceived sleep quality and sleep problems, assessed via self-reports) and quantifiable aspects (e.g., sleep duration and sleep efficiency, assessed via actigraphy). During adolescence, poor sleep health is increasingly recognized as a public health concern. However, there is a lack of evidence on how adolescents’ interactions in contemporary societies, characterized by increasing cultural diversity, are intertwined with sleep health. Thus, this study investigated the reciprocal longitudinal associations between sleep health, considering subjective dimensions (i.e., sleep problems) and quantifiable aspects (i.e., sleep efficiency and duration), and the intercultural interactions of adolescents (quantity and quality) in two different life contexts (i.e., school and leisure time). A total sample of 1470 adolescents living in North-Eastern Italy (*M*_age_ = 15.71, *SD* = 1.22, 47.58% females, 20.58% with a migrant background) wore an actigraph for one week and completed questionnaires about intercultural interactions and sleep health four times across one year. Results of Random-Intercept Cross-Lagged Panel models showed that negative interactions were consistently associated with lower subjective sleep health, lower sleep efficiency, and shorter sleep duration. Conversely, positive intercultural interactions were positively associated with better sleep efficiency, although these effects were mainly observed at the between-person level. These findings underscore the nuanced interplay between the quality of intercultural interactions and both the subjective and objective indicators of sleep health. These findings allow for a conceptualization of adolescents’ sleep as a socially embedded phenomenon shaped by the cultural contexts in which young people live.

## Introduction

Adolescence is a developmental period marked by crucial cognitive, psychological, social, and physical changes with short- and long-term implications for health and well-being (Blakemore, 2019; Meeus, 2016). A core indicator of adolescents’ well-being is their sleep, which is intertwined with many physical and mental health outcomes over time (for review, see Bacaro et al., 2024; Grimaldi et al., 2023). Sleep health is conceptualized as a multidimensional construct (Meltzer et al., 2021), encompassing complementary components, including subjective dimensions (e.g., perceived sleep quality and sleep problems, assessed via self-reports) and quantifiable aspects (e.g., sleep duration and sleep efficiency, assessed via actigraphy).

Nowadays, adolescents’ sleep is considered a public health concern in many countries, as alarming disruptions in both quantity and quality have been observed, as confirmed by both subjective and objective measures (e.g., Carskadon, 2011; Park et al., 2019). This situation can be explained, as theorized in the Perfect Storm Model (Carskadon, 2011; Crowley et al., 2018), on the one hand, by changes in adolescents’ bioregulatory functioning (i.e., a delay in their sleep/wake rhythms) and, on the other hand, by psychosocial factors that characterize their daily life (i.e., self-selected bedtimes, the availability and use of technology and social networking in the evening). The combination of these aspects greatly contrasts societal demands (e.g., early school start times; Afolabi-Brown et al., 2022) and results in the “perfect storm” of short, irregular, and inadequate sleep during adolescence. This can lead to a plethora of poor adjustment outcomes, such as externalizing behavior, internalizing symptoms (Shimizu et al., 2020), and challenges in educational identity formation (Bacaro et al., 2025). Thus, to obtain a comprehensive picture of adolescents’ sleep heath it is necessary to consider its promotion, determinants, and consequences within the broader context in which adolescents are embedded.

Following the socio-ecological framework (Bronfenbrenner, 1979), several social factors were hypothesized to independently or interactively contribute to aspects of youth’s sleep health (Meltzer et al., 2021). Previous research mainly focused on the link between adolescents’ sleep and social relationships in proximal ecological contexts such as peers (for reviews, see De Lise et al., 2023; Gordon et al., 2021). Results highlighted distinct patterns for positive and negative social experiences. Specifically, on the one hand, adolescents’ negative social experiences in their proximal contexts can contribute to poorer sleep health by increasing their stress and demands; on the other hand, disrupted sleep health may hamper adolescents’ ability to cope with stress, negatively affecting their social skills. Conversely, responsive and supportive social experiences with peers may lead to better sleep quality and more consistent sleep patterns, as they foster a sense of emotional security and reduce stress. In turn, adequate and restorative sleep enhances adolescents’ emotional regulation and social competence, promoting more positive and supportive relationships in their everyday interactions. This longitudinal interplay can lead to the potential establishment of vicious or virtuous cycles, significantly impacting adolescents’ psychosocial adjustment (Grandner, 2019). Nevertheless, there has been a notable oversight in the literature regarding how adolescents’ complex social environments and their positive and negative interactions may be intertwined with their sleep health (Bobba et al., 2023).

### Implications of cultural diversity for adolescents’ sleep health

Contemporary societies are characterized by increasing cultural diversity. This growing heterogeneity has important implications for multiple domains of life, including health. Sleep, as a crucial health domain, has been shown to mirror disparities observed in other areas of health inequality, particularly among marginalized groups and ethnic minorities (e.g., Goosby et al., 2018; Yip et al., 2020). Empirical evidence indicates that adolescents from ethnic minorities often exhibit poorer sleep outcomes, including lower sleep efficiency, shorter sleep duration, and greater sleep fragmentation, compared to their peers from the majority groups (Chen et al., 2022; Xie et al., 2021). Regarding these disparities, one prominent explanation centers on the pervasive effects of discrimination and chronic exposure to unfair treatment, which have been consistently linked to impaired sleep health. Mechanisms such as heightened rumination and repetitive negative thinking in response to discriminatory experiences have been proposed as key pathways through which these psychosocial stressors increase cognitive arousal before sleep, hindering nocturnal sleep quality and duration (Arnison et al., 2022; Sladek et al., 2021). Conversely, in both among minority and majority groups, poor sleep health can impair core socio-emotional functions crucial for navigating multicultural interpersonal relationships, heightening emotional reactivity (Hammer et al., 2023) and diminishing empathic abilities (Gordon-Hecker et al., 2025). However, the role of sleep health in adolescence, within the complex relational dynamics of multicultural environments and beyond experiences of discrimination, remains overlooked.

One crucial aspect of increasing cultural diversity is the opportunity to engage in relationships with people from different ethnic groups (Allport, 1954). These intercultural interactions—everyday encounters between individuals from diverse cultural or ethnic backgrounds— can differ not only in frequency but, more importantly, in quality. Recent theoretical developments in intergroup contact research highlight that the quality of contact, whether positive (e.g., warm and respectful) or negative (e.g., unfriendly and intimidating), plays a crucial role in shaping psychosocial aspects (Hayward et al., 2017). Furthermore, the meaning and impact of the quality of these interactions can vary depending on the group to which individuals belong. For example, for people with a migrant background, negative interactions with individuals of Italian origin can be experienced as forms of social marginalization, reflecting structural inequalities between majority and minority groups (Árnadóttir et al., 2022). Consequently, these experiences can be seen as a form of perceived discrimination (Pettigrew & Tropp, 2006). Conversely, when people of Italian origin interact with an individual with a migrant background, the dynamics can be very different. Although these interactions may be unpleasant or hostile, they do not always indicate the same form of structural marginalization experienced by minority groups. Instead, these negative experiences may reflect social prejudices and stereotypes (Albarello et al., 2024). In line with these aspects, positive contact also takes on a different meaning: for people with a migrant background, it can reduce their perception of social marginalization, while for members of the majority group, it can mainly reduce stereotypes and prejudices (Hayward et al., 2017). However, it is essential to note that not all adolescents classified as having a migrant background necessarily perceive themselves as such, and there can be considerable variability even within the same group (Berry, 2009). For example, this variability is reflected in four distinct identity profiles identified among adolescents with a migrant background: ethnicity-oriented identity, nationality-oriented identity, dual identity, and marginalized identity (Karataş et al., 2023a). More specifically, adolescents with an ethnic-oriented identity profile tend to self-identify with their parents' ethnic group and their country of origin. In addition, adolescents with a national identity primarily identify with their destination country. Dual identity, on the other hand, is typical of adolescents who usually perceive both their culture of origin and their destination culture as mutually consistent. Finally, marginalized identity mainly concerns adolescents whose experience of feeling at home does not involve either their parents' country of origin or their destination country. Thus, the meaning and impact of intercultural interactions may differ across individuals, depending on their subjective cultural identification and experiences.

Managing how to navigate this diversity is especially important during adolescence, as youth begin to form and consolidate their approaches to society and others (Bobba & Crocetti, 2022). Generally, adolescents report more positive than negative interactions in their main life contexts (Karataş et al., 2023d). Among them, in the *school context,* they can interact with teachers and classmates from diverse ethnic and cultural backgrounds (Karataş et al., 2023b). *Leisure contexts*, such as participating in sports teams or engaging in informal social activities, also offer valuable opportunities for intercultural interactions with peers from diverse ethnic backgrounds. It is well-known that these interactions have clear implications for adolescents’ intercultural dynamics (e.g., conflict; Al Ramiah & Hewstone, 2013). Specifically, positive interactions can reduce negative attitudes toward others, whereas negative ones are associated with higher levels of prejudice (e.g., Tropp et al., 2022). However, while the impact of intercultural interactions on social attitudes is well documented, their implications for health-related outcomes remain underexplored. To address this gap, it is essential to consider the potential bidirectional relation between intercultural interactions and sleep health.

### The interplay between intercultural interactions and sleep health

The relation between sleep health and intercultural interactions can be understood as a dynamic interplay in which sleep health may influence how individuals navigate social environments and respond to cultural diversity. Specifically, the quality and outcomes of such interactions may depend on individual neurocognitive and emotional regulatory capacities, both of which are closely tied to different aspects of sleep health and can influence adolescents’ social abilities (Immordino-Yang et al., 2025). For instance, aspects of attention, mental flexibility, and working memory are used to support the evaluation of stimuli, particularly useful in multicultural contexts rich in social cues. At the same time, these interactions require the ability to regulate implicit biases, manage social anxiety (Stephan & Stephan, 1985), and promote prosocial behavior toward the "other" (for a review, see Paluck et al., 2021). However, these social-cognitive and emotional processes rely on neurocognitive functioning, which can be negatively impacted by sleep deprivation or disruption (Ben Simon et al., 2018; Killgore, 2010).

In addition, following the “social cure” perspective (Haslam et al., 2018), social experiences can, in turn, impact sleep through emotional and cognitive pathways. Social relationships and group memberships can play a crucial protective role for individuals' health and well-being. Belonging to social groups has been associated with better coping mechanisms and improved physiological outcomes, including better sleep quality (Kent de Grey et al., 2018). In this sense, positive intercultural interactions may act as social resources that enhance sleep health by reducing social isolation and promoting emotional regulation. For instance, positive interactions with others are also linked to fewer physical symptoms (Vang & Nishina, 2022). Conversely, negative intercultural experiences may disrupt identity processes (De Lise et al., 2024) and increase psychological distress, which in turn can influence sleep health (Bobba & Crocetti, 2024). Building on evidence linking adolescents’ intercultural interactions and psychological adjustment, it is important to increase our understanding of how daily interpersonal positive and negative experiences—particularly in intercultural contexts—interact with key health-related processes during adolescence.

### The present study

Moving from these considerations, the present longitudinal study aimed to understand the interplay between sleep health and intercultural interactions in adolescence. The goal is to comprehend how indicators of subjective (i.e., self-reported sleep problems) and objective (i.e., sleep efficiency and duration) sleep health are bidirectionally associated with positive and negative intercultural interactions across two different contexts (school and leisure time). A bidirectional association between the two aspects is expected. Thus, better sleep health (i.e., lower subjective sleep problems, higher sleep efficiency and longer duration) is hypothesized to be associated with more positive interactions in different contexts and vice versa; conversely, lower sleep health (i.e., more subjective sleep problems, lower sleep efficiency and shorter duration) is expected to be bidirectionally associated with more negative interactions across contexts over time. To offer a comprehensive overview, associations were examined to determine whether effects could primarily be explained as (Hamaker et al., 2015) between-person effects which reflect differences across individuals (e.g., do adolescents that generally sleep better than others also report more positive interactions over time?) or within-person effects that capture temporal fluctuations within the same individual (e.g., when an adolescent sleeps worse than usual at a specific time point does report also an increase in negative interactions at a later time?). These associations were examined controlling for adolescents' biological sex, age, and cultural background because these factors were found to be related to adolescents' sleep health (James et al., 2020; Miguez et al., 2020; Saelee et al., 2022) and intercultural interactions (e.g., Karataş et al., 2023b, 2023c, 2023d; Marinucci et al., 2021; Wölfer et al., 2016).

## Method

### Participants

Participants for this study are drawn from the ongoing longitudinal project ‘IDENTITIES: Managing Identities in Diverse Societies: A Developmental Intergroup Perspective with Adolescents. The sample consisted of 1470 adolescents (*M*_age_ = 15.71, *SD*_age_ = 1.22, range: 13.69–20.04 years; 47.58% females, 20.58% with a migrant background). Adolescents were attending the 1st (51.10%) and 3rd (48.90%) year of several high schools (i.e., lyceums 39.51%, technical schools 36.78%, and vocational institutes 23.71%) located in the Northern part of Italy (i.e., the Emilia-Romagna region). These two cohorts were recruited to follow adolescents’ development from 14 to 18 years across the entire secondary school period, which in the Italian school system consists of five years. Among adolescents with a migrant background (i.e., in which at least one parent was not born in Italy), the largest groups had origins in Eastern Europe (33.33%), Africa (24.36%), and Asia (29.49%). Adolescents participated in four assessments across one calendar year (i.e., T1: January/February 2022, T2: April/May 2022, T3: September/October 2022, T4: January/February 2023).

Little (1988) Missing Completely at Random (MCAR) test on all study variables yielded a normed χ2 (χ2/df = 43,853,227/42,022) of 1.04, indicating that data were likely missing completely at random. Regarding the actigraphy recordings specifically, the proportions of adolescents who provided fewer than 3 nights of data were 4.57% at T1, 11.82% at T2, 32.21% at T3, and 31.23% at T4. Accordingly, across the four waves, the majority of participants provided at least three nights of data (i.e., 89.47% of the sample). Therefore, the total sample of 1,470 adolescents was included in the analyses, and missing data were handled with the Full Information Maximum Likelihood (FIML) procedure available in M*plus* (Kelloway, 2015).

### Procedure

This longitudinal research was approved by the Ethics Committee of University of Bologna as part of the project ‘IDENTITIES: Managing Identities in Diverse Societies: A Developmental Intergroup Perspective with Adolescents’. Schools were selected through a stratified, randomized method (by track and level of urbanization), and principals were approached to present the project. Then, the study was presented to students and their parents, who received detailed oral and written information. Active parental consent was obtained prior to participation. Active consent was also obtained from adolescents of age, while their underage peers provided their assent to participate in the project. Participation in the study was voluntary, and students were informed that they could withdraw their consent or assent at any time. At each wave, adolescents first received an actigraph to Nwear for seven days and seven nights. On the eighth day, they completed an online questionnaire during school hours. Research assistants were present in the class to answer any questions students had. Adolescents had to create a personal code to ensure confidentiality and pair their answers over time and across assessment methods.

### Measures

#### Demographics

Participants’ socio-demographic information (i.e., biological sex, age, and cultural background) was collected at T1, while other measures described below were collected at all four time points.

#### Subjective sleep problems

Problems of the sleep/wake cycle were assessed with the sleep subscale of the Mini Sleep Questionnaire (MSQ; Zomer et al., 1985; Italian validation by Natale et al., 2014). This subscale consists of five items (e.g., “Did you have troubles in falling asleep?”) rated on 7-point Likert type scales from 1 (*never*) to 7 (*always*). Higher scores indicate greater sleep problems. Cronbach’s alphas are reported in Table 1.

**Table 1:** Cronbach’s alphas (α), means (M), and standard deviations (SD) of study variables.

	T1			T2			T3			T4		
	α	*M*	*SD*	α	*M*	*SD*	α	*M*	*SD*	α	*M*	*SD*
Sleep Health												
Subjective sleep problems	.74	2.63	1.09	.79	2.72	1.23	.80	2.57	1.20	.79	2.60	1.13
Objective sleep efficiency		92.33 %	3.40		91.62%	3.79		92.72%	3.65		92.75%	3.40
Objective sleep duration		7.08 h	0.74		6.84 h	0.82		7.02 h	0.82		6.94 h	0.81
Intercultural interactions at school												
Positive	.93	3.98	0.72	.94	4.00	0.71	.94	3.99	0.71	.93	3.90	0.73
Negative	.84	1.64	0.69	.88	1.76	0.80	.88	1.76	0.77	.90	1.88	0.82
Intercultural interactions during leisure time												
Positive	.93	3.94	0.75	.94	3.93	0.72	.95	3.86	0.76	.91	3.81	0.71
Negative	.87	1.69	0.72	.91	1.81	0.83	.92	1.87	0.83	.91	1.96	0.84

#### Objective sleep

To evaluate the quantifiable aspects of sleep, the following actigraphic sleep parameters were considered: sleep duration represented by total sleep time minutes (i.e., the sum of all sleep epochs between sleep onset and sleep offset, with the first epoch of the first 20-min block of persistent sleep, with no more than 1 min of wake), and sleep efficiency (i.e., the ratio between total sleep time and the total time individuals spend in bed, multiplied by 100). In the present study, at each assessment, adolescents wore a wrist actigraph (Micro Motionlogger Watch, Ambulatory Monitoring, Inc.; Ardsley, NY) for seven consecutive days and nights. The actigraph was initialized in zero-crossing mode through the Motionlogger Watchware software (Ambulatory Monitoring, Inc., Ardsley, NY, USA) to collect data in 1-min epochs. Adolescents were instructed to wear the actigraph continuously on their non-dominant wrist and to remove it only during contact sports or while showering to prevent damage. In addition, they were asked to press the event marker on the actigraph to indicate when they (a) turned off the lights to go to sleep at night and (b) got out of bed in the morning. The actigraphic data were analyzed through Action W-2® software, version 2.7.3285 (Ambulatory Monitoring, Inc., Ardsley, NY), using previously validated algorithms (Cole & Kripke, 1988; Cole et al., 1992). To be included in the analysis, actigraphic records should report at least one valid night of recorded activity.

#### Adolescents’ intercultural interactions at school/during leisure time

Participants reported the quantity and quality of their intercultural interactions by filling the Intergroup Contact Interactions Scale (ICIS; Karataş et al., 2023d), a questionnaire developed and validated for the Italian language. Italian adolescents were asked to reflect on interactions with people of foreign origin (i.e., people with a cultural background different from their own); conversely, adolescents with a migrant background were asked to reflect on interactions with people of Italian origin. Participants were asked: “In the last 4 months, at school (e.g., with other students, teachers)/during leisure time (e.g., your town, neighborhood), have you met and talked with people of Italian/foreign origin?” Then, they evaluated their interactions by answering 10 items: five for positive interactions (e.g., “They have been polite to you”) and five for negative interactions (e.g., “They have been rude to you”). The quality of these interactions was rated on a 5-point Likert scale from 1 (*never*) to 5 (*very often*). Cronbach’s alphas are reported in Table 1.

#### Strategy of analysis

Descriptive statistics, normality indices of variables, correlations, and reliability analyses were conducted in IBM SPSS Version 29.0 for Windows. In addition, to explore potential group differences, repeated-measures ANOVAs were also conducted in IBM SPSS Version 29.0 for Windows, with cultural background (Italian adolescents vs. adolescents with a migrant background) as the between-subjects factor and time (T1, T2, T3, T4) as the within-subjects factor. All other analyses were conducted in Mplus 8.11 (Muthén & Muthén, 2017) using the Maximum Likelihood Robust (MLR) estimator (Satorra & Bentler, 2001). The longitudinal measurement invariance for subjective sleep problems and intercultural interactions at school and during leisure time was tested as a preliminary step. Specifically, to establish metric (i.e., constraining factor loadings to be equal across time) and scalar (i.e., constraining intercepts to be equal across time) invariance, changes in fit indices from one model to the next (i.e., from the configural to the metric, and from the metric to the scalar) were evaluated. Then, Four Random Intercept – Cross Lagged Panel Models (RI‐CLPM, Hamaker et al., 2015) were performed to test the main hypotheses. Two models investigated associations between sleep health and intercultural interactions in the school context: one examined the association between intercultural interactions (i.e., positive and negative) and subjective sleep problems, and the other examined associations with objective sleep efficiency and duration. The other two modelled the same associations in the context of leisure time. RI-CLPMs allowed the analysis of associations over time between sleep health and intercultural interactions, disentangling between-person and within-person effects. In all models, adolescents' biological sex, age, and cultural background were added as covariates. An unconstrained model (M1) was initially tested to estimate cross-lagged paths, controlling for stability paths (T1→T2, T2→T3, and T3→T4) and within-time correlations among all study variables at each time point. To establish the model as parsimonious as possible, alternative models (M2) with cross-lagged paths constrained to be equal across time (e.g., T1→T2 cross-lagged paths fixed to be equal to T2→T3 paths) were estimated and compared to the baseline model (M1). Afterward, models (M3) in both cross-lagged and correlations at T2, T3, and T4 were constrained to be equal and were tested and compared to the previous model (M2). Next, models (M4) with cross-lagged, correlated changes, and autoregressive paths fixed to be equal across time points were compared against the previous ones (M3). Finally, a model (M5) with the effects of covariates constrained to be equal across time was estimated and compared to the former model (M4).

Fit indices for longitudinal measurement invariance and model comparisons of RI-CLPMs were evaluated based on the following criteria. The Comparative Fit Index (CFI) with values above 0.90 and 0.95, respectively, indicate acceptable and very good fit. The Root Mean Square Error of Approximation (RMSEA) and the Standardized Root Mean Residual (SRMR) with values below 0.08 and 0.05, respectively, indicate acceptable and very good fit (Byrne, 2012). Additionally, the RMSEA’s 90% confidence interval upper bound is below 0.10, indicating an acceptable fit of the model (Chen et al., 2008). To establish invariance, changes in fit indices across models were evaluated using multiple indicators (e.g., Cheung & Rensvold, 2002). Specifically, a significant ΔχSB2 (Satorra & Bentler, 2001), and ΔCFI ≥ −0.010 supplemented by ΔRMSEA ≥ 0.015 (Chen, 2007) are indicative of non-invariance.

## Results

### Preliminary results

The means and standard deviations of study variables at T1, T2, T3, and T4 are reported in Table 1, while the normality indices of study variables are reported in Supplemental Materials (see Table S1). As shown in Table 1, adolescents reported, on average, sleep problem scores below the scale midpoint, suggesting low levels of sleep difficulties. Additionally, their average objective sleep efficiency was 92.36%, indicating good sleep quality, in line with the thresholds recommended by the National Sleep Foundation (i.e., ≥ 85%; Ohayon et al., 2017). Regarding objective sleep duration, adolescents reported an average total sleep time of 6.97 h across the four waves, thus below the recommended range (i.e., 8–10 h per night; Hirshkowitz et al., 2015). Furthermore, adolescents reported more positive than negative intercultural interactions in both contexts.

The results of a repeated-measures ANOVA are reported in Table 2. Overall, adolescents with a migrant background reported higher levels of subjective sleep problems and lower objective sleep efficiency compared to their Italian peers. These differences were statistically significant, although the effect sizes were small. No significant group differences were observed in either context for sleep duration or intercultural interactions.

**Table 2:** Repeated measure analyses to compare study variables across time points in relation to adolescents’ cultural background.

Mean-level changes							
		M_T1_*(SD)*	*M_T2_ (SD)*	*M_T3_ (SD)*	*M_T4_(SD)*	*d*	*F-test (η^2^)*
Subjective sleep problems	Italian adolescents	2.52 (1.01)	2.58 (1.12)	2.48 (1.14)	2.51 (1.08)	0.20	5.065* (0.009)
	Adolescents with a migrant background	2.75 (1.16)	2.80 (1.28)	2.88 (1.25)	2.70 (1.11)		
Objective sleep efficiency	Italian adolescents	92.69% (3.06)	92.05% (3.44)	92.76% (3.72)	93.25% (3.11)	0.24	1.817 (0.179)
	Adolescents with a migrant background	91.90% (3.23)	91.50% (3.95)	92.13% (3.61)	92.50% (3.12)		
Objective sleep duration	Italian adolescents	7.17 h (0.69)	6.95 h (0.73)	7.12 h (0.70)	7.04 h (0.74)	0.31	5.570* (0.018)
	Adolescents with a migrant background	6.89 h (0.84)	6.70 h (0.79)	6.86 h (0.89)	6.77 h (0.98)		
Positive interactions at school	Italian adolescents	4.02 (0.68)	4.10 (0.66)	4.07 (0.64)	3.97 (0.69)	0.19	0.856 (0.003)
	Adolescents with a migrant background	4.07 (0.60)	4.13 (0.67)	4.18 (0.75)	4.04 (0.75)		
Negative interactions at school	Italian adolescents	1.55 (0.61)	1.66 (0.69)	1.67 (0.68)	1.77 (0.79)	0.28	2.780 (0.009)
	Adolescents with a migrant background	1.74 (0.60)	1.80 (0.75)	1.77 (0.72)	1.87 (0.82)		
Positive interactions during leisure time	Italian adolescents	3.97 (0.69)	3.97 (0.66)	3.92 (0.69)	3.91 (0.71)	0.17	1.401 (0.010)
	Adolescents with a migrant background	4.03 (0.58)	4.08 (0.69)	4.19 (0.65)	4.03 (0.68)		
Negative interactions during leisure time	Italian adolescents	1.67 (0.72)	1.76 (0.78)	1.79 (0.77)	1.86 (0.83)	0.18	0.457 (0.003)
	Adolescents with a migrant background	1.56 (0.57)	1.75 (0.81)	1.69 (0.72)	1.72 (0.71)		

*Note. M* = Mean, SD = Standard Deviation, *F-test*= Fisher’s coefficient, *η2*= partial eta squared, *d* = Cohen’s d. Significant F effects are highlighted in bold.

⁎ *p* < .05, ^⁎⁎^*p* < .01, ^⁎⁎⁎^*p* < .001

Correlations between the main variables are available in the Supplemental Materials (see Tables S2, S3, S4, S5). Results indicated small associations between study variables. Finally, metric longitudinal invariance was established for all models, as detailed in the Supplemental Materials (see Table S6). Thus, the observed variables could be used to test the main hypotheses.

### Random intercept – cross lagged panel models

**Fig. 1:**
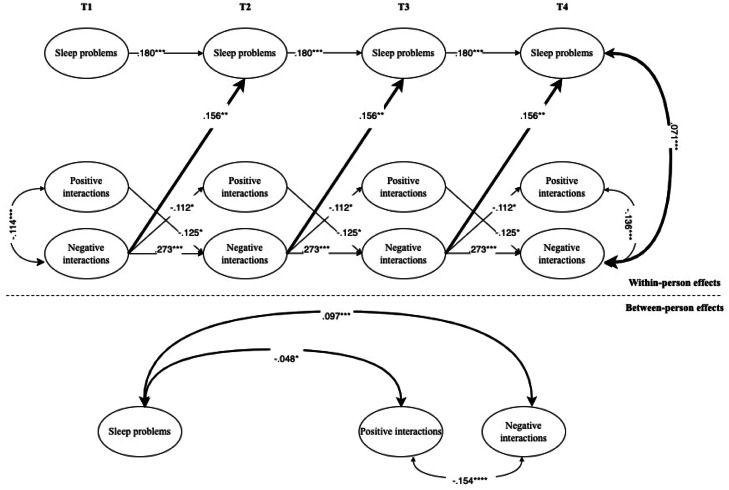
Interplay between adolescents’ subjective sleep problems and intercultural interactions at school. *Note.* For the sake of clarity, only significant standardized coefficients are displayed. Subjective sleep problems have been coded with higher values indicative of more problems. Intercultural interactions have been coded with higher values indicative of higher positive or negative intercultural interactions. In the RI-CLPM shown, the following parameters were constrained to be equal across waves: covariances with covariates, autoregressive (stability) paths, cross-lagged paths, and correlated residuals. Bold arrows indicate significant effects between adolescents’ sleep problems and interethnic interactions. Black arrows indicate within-construct effects (e.g., paths between positive and negative interethnic interactions of adolescents). * *p* < .05; ^⁎⁎^*p* < .01; ^⁎⁎⁎^*p* < .001.

**Fig. 2:**
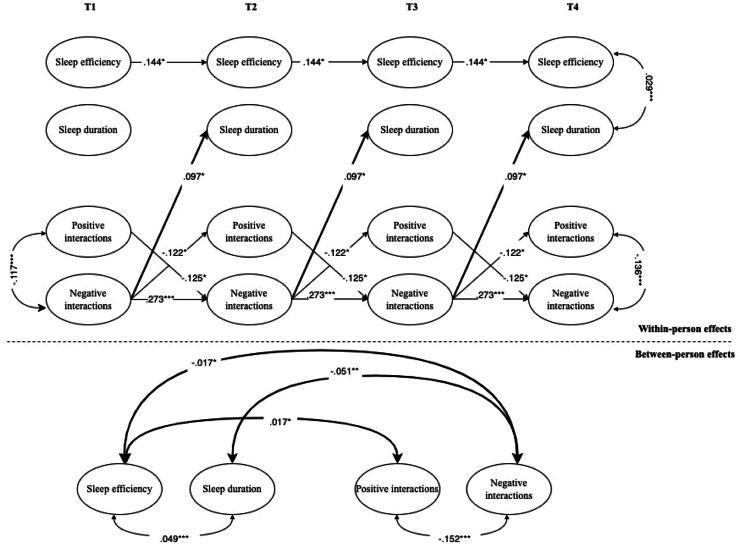
Interplay between adolescents’ objective sleep and intercultural interactions at school. *Note.* For the sake of clarity, only significant standardized coefficients are displayed. Sleep efficiency have been coded with higher values indicative of more time spent sleeping and high levels of efficiency, respectively. Intercultural interactions have been coded with higher values indicative of higher positive or negative intercultural interactions. In the RI-CLPM shown, the following parameters were constrained to be equal across waves: covariances with covariates, autoregressive (stability) paths, cross-lagged paths, and correlated residuals. Bold arrows indicate significant effects between adolescents’ sleep problems and interethnic interactions. Black arrows indicate within-construct effects (e.g., paths between positive and negative interethnic interactions of adolescents). * *p* < .05; ^⁎⁎^*p* < .01; ^⁎⁎⁎^*p* < .001.

**Fig. 3:**
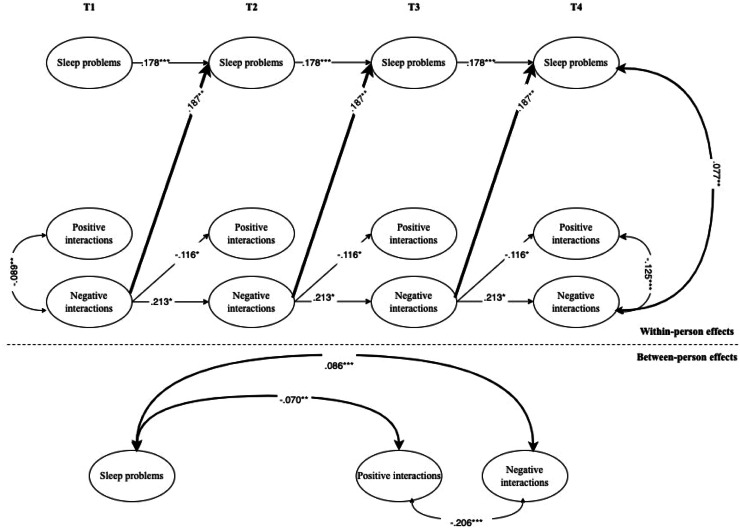
Interplay between adolescents’ subjective sleep problems and intercultural interactions during leisure time. *Note.* For the sake of clarity, only significant standardized coefficients are displayed. Subjective sleep problems have been coded with higher values indicative of more problems. Intercultural interactions have been coded with higher values indicative of higher positive or negative intercultural interactions. In the RI-CLPM shown, the following parameters were constrained to be equal across waves: covariances with covariates, autoregressive (stability) paths, cross-lagged paths, and correlated residuals. Bold arrows indicate significant effects between adolescents’ sleep problems and interethnic interactions. Black arrows indicate within-construct effects (e.g., paths between positive and negative interethnic interactions of adolescents). * *p* < .05; ^⁎⁎^*p* < .01; ^⁎⁎⁎^*p* < .001.

**Fig. 4:**
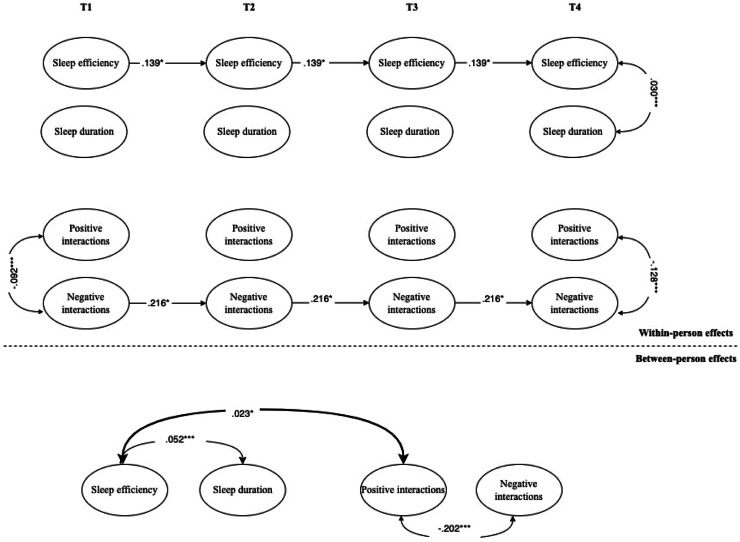
Interplay between adolescents’ objective sleep and intercultural interactions during leisure time. *Note.* For the sake of clarity, only significant standardized coefficients are displayed. Sleep efficiency has been coded with higher values indicative of more time spent sleeping and high levels of efficiency, respectively. Intercultural interactions have been coded with higher values indicative of higher positive or negative intercultural interactions. In the RI-CLPM shown, the following parameters were constrained to be equal across waves: covariances with covariates, autoregressive (stability) paths, cross-lagged paths, and correlated residuals. Bold arrows indicate significant effects between adolescents’ sleep problems and interethnic interactions. Black arrows indicate within-construct effects (e.g., paths between positive and negative interethnic interactions of adolescents). * *p* < .05; ^⁎⁎^*p* < .01; ^⁎⁎⁎^*p* < .001.

Model comparison, available in the Supplemental Materials (see Table S7), established that the most parsimonious models (M4) fitted the data well and could be retained as the final models. Across the four models, stability paths were significant for all variables, except for positive interactions. Detailed results are reported below and in Fig. 1, Fig. 2, Fig. 3, Fig. 4.

#### Longitudinal associations between sleep health and intercultural interactions in the school context

**Subjective sleep problems.** Regarding the associations between subjective sleep problems and intercultural interactions in the school context, the within-person results of the RI-CLPM (see Fig. 1) showed that adolescents who reported more negative intercultural interactions at school also reported higher sleep problems at subsequent time points. This result was confirmed by within-person correlated changes, where adolescents’ sleep problems were associated with more negative interactions at T2, T3, and T4. At the between-person level, sleep problems were positively related to negative interactions while negatively linked to positive interactions.

**Objective sleep.** Concerning the longitudinal association between objective sleep efficiency and intercultural interactions in the school context, within-person results of the RI-CLPM (see Fig. 2) did not show any significant associations. Instead, regarding objective sleep duration, a small positive association over time was observed with negative interactions. Furthermore, at the between-person level, sleep efficiency was positively associated with positive interactions and negatively associated with negative interactions. In addition, sleep duration was negatively associated with negative intercultural interactions.

#### Longitudinal associations between sleep and intercultural interactions during leisure time

**Subjective sleep problems.** Regarding the associations between subjective sleep problems and intercultural interactions in the leisure-time context, the within-person results of the RI-CLPM (see Fig. 3) showed that individuals who reported more negative interactions also showed higher levels of sleep problems. This result was partially confirmed by within-person correlated changes, in which higher levels of sleep problems were associated with more negative interactions at T2, T3, and T4. At the between-person level, sleep problems were positively related to negative interactions and negatively related to positive interactions.

**Objective sleep.** As for the longitudinal association between objective sleep efficiency and duration, and intercultural interactions during leisure time (Fig. 4), at the within-person level, no significant associations over time or within-time correlations emerged. Nevertheless, at the between-person level, sleep efficiency was positively associated with positive interactions, whereas sleep duration showed no significant association.

#### Covariates

The results reported above were obtained by controlling for sex, age, and cultural background. Findings related to the covariates are available in Table 3. Biological sex was associated with adolescents' sleep health (i.e., girls reported more subjective sleep problems but higher objective sleep efficiency and longer sleep duration than boys) and quality of interactions in both contexts (i.e., girls reported more positive interactions than boys, who reported more negative interactions). Also, cultural background was associated with sleep health and intercultural interactions: adolescents with a migrant background reported lower objective sleep efficiency than their Italian peers, as well as a higher number of interactions in both contexts, with a tendency toward more positive interactions during leisure time and more negative interactions at school. In addition, age was negatively associated with sleep duration, indicating that older adolescents tend to sleep for shorter durations, whereas age was unrelated to the other study variables.

**Table 3:** Covariates effects on study variables.

		Covariates		
		Biological sex (*β*)	Age (*β*)	Cultural background (*β*)
Sleep health	Subjective sleep problems	.274⁎⁎⁎	.045	.081
	Objective sleep efficiency	.352⁎⁎⁎	.023	−0.120⁎⁎
	Objective sleep duration	.169⁎⁎⁎	−0.290⁎⁎⁎	.030
Intercultural interactions at school	Positive interactions	.198⁎⁎⁎	−0.038	.066
	Negative interactions	−0.125⁎⁎⁎	.030	.114⁎⁎⁎
Intercultural interactions during leisure time	Positive interactions	.170⁎⁎⁎	−0.045	.120⁎⁎⁎
	Negative interactions	−0.155⁎⁎⁎	.058	.014

*Note. β*= Standardized Beta. Sex (0 = boys, 1 = girls); Age (0= adolescents enrolled in the first year of school at T1, 1= adolescents enrolled in the third year of school at T1); Cultural background (0= Italian adolescents, 1= adolescents with a migrant background). For the sake of clarity, the standardized beta coefficients for sleep variables are reported as the mean between the four time points (T1-T4) and between school and leisure time models, in order to minimize redundancy. In the same way, since the effects on positive and negative intercultural interactions in school and leisure time contexts were very similar in the subjective and objective sleep models, the coefficients were averaged between the school and leisure time models, and between T1-T4. Significant effects are indicated in bold.

⁎⁎ *p* < .01.

⁎⁎⁎ *p* < .001.

## Discussion

Adolescence is a developmental period marked by significant changes across multiple levels, including psychophysiological processes related to the social contexts in which adolescents are embedded (Meeus, 2016). These developmental changes can disrupt sleep patterns (Carskadon, 2011; Crowley et al., 2018) and compromise sleep health, which is closely linked to adolescents’ well-being (for a review, see Bacaro et al., 2024). Indeed, adolescents are embedded in complex social environments shaped by the challenges of contemporary societies, which may also influence their sleep health (Bobba et al., 2023). While it is well-known that these interactions have clear implications for adolescents’ intercultural dynamics (e.g., conflict; Al Ramiah & Hewstone, 2013) and can foster or hinder their adjustment, less is known about how such interactions are bidirectionally associated with other key aspects of their well-being, such as their sleep health. Thus, the present study investigated the reciprocal longitudinal connections between adolescents’ sleep health (including subjective sleep problems and objective sleep efficiency and duration) and the quality of intercultural interactions in two different contexts (i.e., school and leisure time). Within-person and between-person effects were disentangled, underscoring distinct patterns of associations for each level.

### Within-person effects: the costs of negative intercultural interactions for sleep health

Contrary to the hypothesis, it was found that intercultural interactions mainly influenced sleep health, not vice versa. Specifically, results showed that individuals who have more negative intercultural interactions also displayed worse subjective sleep health (i.e., higher sleep problems) over time in both contexts. This result emerged from cross-lagged effects and was further corroborated by significant temporal correlations.

These results highlighted a stronger negative than positive effect on adolescents’ sleep health. They can be interpreted in line with the valence-salience effect (Paolini et al., 2010), which holds that negative interactions have greater psychological salience and impact than positive ones. This asymmetry has been observed across psychological domains, such as memory (Kensinger, 2009) and social cognition (Agadzhanyan & Castel, 2024), in which negative events tend to have stronger effects than positive ones (Baumeister et al., 2001). Thus, regarding intercultural relations, even occasional negative interactions are more likely to evoke stronger emotional responses that can influence well-being indicators, such as sleep health.

This evidence can also be further explained considering stress-response mechanisms. Specifically, negative intercultural interactions can act as psychosocial stressors that activate physiological arousal, disrupting sleep patterns (Tang & Harvey, 2004). In adolescents, greater stress reactivity is typically associated with a decline in sleep health, especially regarding subjective sleep problems (Kater et al., 2022), consistent with the present study. Additionally, sleep problems and negative interactions were associated. Thus, the nocturnal consequences of sleep problems can be associated with lower-quality social interactions. Sleep-based changes in emotional reactivity are rooted in disrupted emotion regulation (Gross, 1998; Tarokh et al., 2016), with disrupted sleep resulting in greater dysregulated emotion, which has been identified as a potential key mechanism linking sleep and social functioning (e.g., Tavernier & Willoughby, 2015).

Finally, it is worth noting that these results were not confirmed for the objective components of sleep. No significant cross-lagged paths emerged for sleep efficiency and intercultural interactions, while a small within-person association was observed with sleep duration. Specifically, in the school context, occasions when adolescents experienced more negative intercultural interactions than typical were associated with slightly longer sleep duration. This within-person effect, which reflects temporal fluctuations around each individual’s average, might capture short-term changes in adolescents’ behavioral responses to stress (Green & McCormick, 2013). For instance, when experiencing greater interpersonal tension, some adolescents may temporarily spend more time sleeping to disengage or recover from daily strain, without necessarily improving sleep quality. Indeed, research suggests that sleep duration and sleep quality can show distinct patterns in relation to stress exposure, with compensatory increases in time spent asleep not always translating into restorative sleep (e.g., Dewald et al., 2010; Wang & Yip, 2020). Overall, these results confirmed the importance of distinguishing between the subjective and objective components of sleep health (Bobba & Crocetti, 2024). For instance, subjective and objective indicators of sleep health do not always show consistent associations, and their relationship can vary considerably across studies. This suggests that these indicators may capture partially distinct aspects of sleep. Objective measures provide valuable information about quantifiable dimensions of sleep (e.g., duration, efficiency), yet they may not fully reflect the subjective experience of sleep problems. Conversely, self-reported indicators, such as perceived sleep problems, are often shaped by emotional and cognitive factors, including mood, stress, and expectations about one’s sleep (Cudney et al., 2022), thereby reflecting a different, though equally important, facet of sleep health.

Considering both subjective and objective measures thus allows for a more comprehensive understanding of sleep functioning. Indeed, adolescents may exhibit adequate sleep efficiency from a physiological perspective while perceiving their sleep as poor due to various factors (Tremaine et al., 2010). Nevertheless, subjective perception can have real consequences on daytime functioning, independent of objective sleep parameters, and impact adolescents’ adjustment (Lucas-Thompson et al., 2021). This supports the notion that adolescents’ subjective experiences of sleep are meaningful, as they can influence emotional well-being and social functioning even when they are not aligned with objective indicators. However, the observed link between subjective sleep problems and perceptions of intergroup contact could also be partly driven by a broader dispositional tendency—such as a general negative bias toward oneself and one’s social environment. Individuals characterized by a more negative interpretative style may perceive both their sleep and their social experiences negatively, thereby inflating associations between the two (Puccetti et al., 2023).

### Between-person effects: the interplay of intercultural interactions and sleep health

Moving to the between-person level, results corroborated findings discussed at the within-person level while also further expanding them. Specifically, the association between negative interactions and sleep problems found at the within-person level was replicated in both contexts. Additionally, a significant link emerged between higher levels of negative interactions in the school context and lower objective sleep efficiency and shorter sleep duration. Regarding the positive interactions, consistent with the within-level findings, results showed that higher levels of positive interactions were associated with lower sleep problems (in the school context only) and higher sleep efficiency (across both contexts), but not with sleep duration.

These results offer meaningful insights into how stable patterns of intercultural interactions relate to adolescents' sleep health, capturing broader interindividual differences. This suggests the importance of considering sleep health as a multifaceted construct that can be assessed with both subjective and objective measures, and that may influence adolescents’ capacity to engage in intercultural relationships through different pathways, thereby extending prior evidence on the role of peers (De Lise et al., 2023). The associations between negative peer relationships and sleep efficiency and duration aligned with existing research indicating that poor sleep influences socio-emotional functioning, such as emotional regulation and empathy, which are essential for managing social interactions (Guadagni et al., 2014; Palmer & Alfano, 2017). In turn, the experience of negative interactions may lead to sleep disruption, including objective measures of sleep initiation and maintenance during the night. Although discrimination was not directly assessed in this study, the observed link between objective sleep health and the quality of intercultural interactions may, at least in part, reflect stress-related processes common in multicultural environments. Especially adolescents with a migrant background often experience nocturnal sleep difficulties—due to psychosocial stressors like discrimination—which can disrupt sleep through mechanisms such as rumination and hyperarousal (e.g., Sladek et al., 2021).

Second, while negative experiences appear to compromise sleep through stress-related mechanisms, it is equally important to consider that the association between positive interactions and sleep health consistently emerged. Specifically, adolescents who are more engaged in positive intercultural interactions, compared to those with fewer positive interactions, can also have better sleep health (i.e., lower sleep problems and higher sleep efficiency), and vice versa over time. A possible explanation is that between-person effects capture stable, trait-like differences between individuals—such as a general tendency to experience more positive social interactions or maintain healthier sleep patterns—while within-person effects reflect short-term, state-like fluctuations around an individual’s own average (Hamaker et al., 2015). Overall, these findings emphasize that in line with the "social cure" perspective (Haslam et al., 2018), social relationships and group memberships can play a crucial role in shaping individuals' well-being—not only by providing emotional support but also by mitigating the negative impact of stressors such as discrimination. Thus, fostering positive intercultural interactions in multicultural contexts may be valuable for promoting social integration and psychological resilience, particularly among adolescents navigating complex social environments.

### The interplay between sleep health and intercultural interactions: how age, biological sex, and cultural background matter

The present results were obtained by controlling for adolescents' age, biological sex, and cultural background. Regarding age, consistent with previous research (e.g., Marinucci et al., 2021; Park et al., 2019), older adolescents reported shorter sleep duration. This finding aligns with the developmental phase, as sleep duration typically decreases across adolescence due to normative changes in adolescents’ sleep patterns (Blakemore, 2019; Meeus, 2016). In contrast, no differences emerged in the other aspects of sleep health (i.e., sleep problems and sleep efficiency) or in intercultural interactions.

Regarding biological sex, despite reporting more subjective sleep problems, girls exhibited higher objective sleep efficiency and sleep duration compared to boys. The finding is in line with previous research, which showed that females often perceive greater sleep difficulties even when objective measures suggest relatively better sleep quality (Carter et al., 2020). This discrepancy underscores the importance of considering both subjective and objective assessments in sleep research (Trimmel et al., 2021). Furthermore, girls reported more positive interactions than boys across both school and leisure time contexts. These findings reflect females' tendency to engage in more pleasant and emotionally supportive social interactions (e.g., Karataş et al., 2023b). Such patterns may be attributed to gender socialization processes that encourage girls to develop greater empathy and interpersonal sensitivity (Rochat, 2023), which in turn can foster more harmonious social experiences.

Finally, cultural background also significantly affected sleep health and intercultural interactions. Specifically, adolescents with a migrant background reported lower objective sleep efficiency compared to their Italian peers. This result may be explained by the social stressors they often face, such as acculturative pressure, perceived discrimination, and socioeconomic challenges, which have been shown to disrupt sleep regulation and contribute to increased sleep problems (e.g., Huynh & Gillen-O’Neel, 2016; Yip et al., 2020). Moreover, adolescents with a migrant background engaged in more negative interactions at school and more positive interactions during leisure time. This finding may reflect the social challenges these adolescents face, as they often navigate complex intercultural dynamics within schools. Still, they can find social support in unstructured peer contexts (e.g., Verkuyten & Martinovic, 2017). Overall, these results highlighted the role of sociodemographic factors in shaping adolescents’ social interactions and sleep health, emphasizing the need for culturally sensitive approaches in both research and intervention.

### Limitations and future research directions

Starting from the notable strengths of the present study, the following section outlines its key advantages and subsequently discusses the main limitations to be considered when interpreting the findings. First, the study was conducted in naturalistic settings to capture adolescents’ health patterns in everyday life. This ecological approach strengthens the external validity of the findings. According to actigraphy guidelines (Littner et al., 2003), participants were instructed to wear the actigraph continuously for one week at each assessment, removing it only during contact sports or showering. Importantly, the key requirement was to ensure continuous wear for at least three consecutive 24-h periods, which is considered sufficient to capture stable sleep–wake patterns in naturalistic settings. However, given the large community-based sample, adherence to these instructions varied, leading to unequal numbers of valid nights across participants. To maintain inclusiveness and ecological validity, participants with at least one valid night of actigraphic data per assessment were retained, acknowledging that this may reduce the reliability of individual estimates. Although adolescents who wore the actigraph for at least three nights across the four waves accounted for the majority of the sample (i.e., 89.28%), this methodological compromise should nevertheless be considered when interpreting the results. Considering that adolescents’ sleep patterns often vary considerably across the week, particularly between school days and weekends (i.e., social jet lag; Tonetti et al., 2023), future studies could benefit from longer monitoring periods and a greater number of valid nights that systematically capture both weekday and weekend sleep patterns, thereby providing more robust and generalizable estimates of adolescents’ sleep health in naturalistic settings.

Second, by examining the longitudinal associations between the quality of intercultural interactions and adolescents’ sleep health, the study provides valuable insights. However, it does not address the mechanisms that might explain these associations. Future studies should expand on these results to examine potential mediators that clarify whether intercultural experiences affect sleep health directly or indirectly through psychological, emotional, or social processes. In line with this, studies could examine adolescents’ interactions with people of the same cultural background. Including this control variable would allow researchers to examine interactions with both in-group (one’s own cultural group) and out-group (people from different cultural groups) members, helping to disentangle the specific influence of intercultural experiences from adolescents’ general tendency to perceive social interactions positively or negatively. Addressing these aspects would strengthen the validity of future findings and provide a clearer understanding of how culture shapes adolescents’ sleep health.

Third, the study offers an overview of the interplay between sleep health and intercultural interactions across adolescents with and without a migrant background. Specifically, in line with common practice (Chang et al., 2022; Kajikhina et al., 2023), adolescents were classified as having a migrant background based on their own or their parents’ country of birth. However, this measure does not consider how people actually perceive and express their cultural belonging (Berry, 2009). For example, some adolescents, despite having a migrant background, may feel fully Italian, while others may maintain strong ties to multiple cultural contexts (Karataş et al., 2023a). In addition, since the majority of participants did not have a migrant background, the observed associations are mainly driven by Italian adolescents’ interactions with peers of migrant origin, and moderation effects could not be reliably tested. Thus, future research should consider cultural background as a potential moderator, along with other factors such as social support. For instance, daily support from important figures (e.g., parents, friends, and teachers) may moderate the relationship between intercultural interactions and sleep health (Chen et al., 2022). Investigating these additional interpersonal or cultural factors as potential moderators could further clarify the conditions under which sleep health and intercultural interactions are more intertwined.

Finally, a key strength of the present study is its longitudinal design, which included four assessments over the course of one year, allowing for the examination of changes in adolescents’ sleep health and intercultural interactions over time. Nevertheless, the relatively short duration of the present study may have constrained the variability in intercultural interactions, potentially limiting the power to detect bidirectional effects between interactions and sleep health. Moreover, the quality of intercultural interactions and sleep patterns may be susceptible to ongoing change in response to daily experiences (Graf et al., 2014; Matos et al., 2016). To address these limitations, future research could combine longer-term longitudinal designs with intensive daily measurements to capture both gradual developmental changes and short-term fluctuations. Such an approach would provide a more comprehensive understanding of the dynamic interplay between adolescents’ intercultural interactions and sleep health, shedding light on how psychosocial and environmental factors influence sleep over time and on a daily basis.

## Clinical implications

Overall, these results highlight the importance of acknowledging adolescents’ embeddedness in increasingly diverse societies and of emphasizing the potential role of intercultural interactions as factors contributing to their well-being. This perspective is particularly relevant for informing future evidence-based interventions fostering both sleep health and intercultural interactions within a broader psychosocial framework. Specifically, the association between negative intercultural interactions and sleep problems suggests that interventions should incorporate psychological components aimed at enhancing emotion regulation, stress coping, and social skills. For instance, cognitive-behavioral interventions (CBT-I), recognized as the gold standard treatment for insomnia (Riemann et al., 2023), can be adapted to not only intervene when insomnia is diagnosed but also to target adolescents sleep health in general and to integrate aspects that focus not only on sleep hygiene (i.e., sleep psychoeducational programs in the school context), but also on improving peer relationships and intercultural competence. Tailored approaches that consider both physiological and psychosocial factors contributing to sleep problems (e.g., discrimination or acculturative stress) can more effectively support the well-being and adaptation of adolescents living in multicultural societies.

Additionally, clinicians, practitioners, and teachers should consider how adolescents’ different life contexts (e.g., school and leisure time) play distinct roles in shaping sleep health and social experiences, with important implications for prevention programs. Within the school context, teachers’ involvement can foster a positive climate (Maratia et al., 2025) that supports constructive peer interactions and reduces stressors that negatively impact sleep health. Furthermore, schools offer a structured environment in which psychoeducational programs can be systematically implemented (Crocetti et al., 2025), promoting awareness of healthy sleep habits alongside social-emotional learning. In less structured leisure contexts, such as sports teams, programs can leverage the voluntary nature of participation to promote sleep hygiene and coping strategies, even among culturally diverse groups. Importantly, both school settings and leisure contexts can serve as key platforms for interventions that address sleep and well-being, highlighting the potential positive outcomes of living in multicultural environments. Such interventions can focus on supporting adolescents in navigating cultural identity and diversity (Crocetti et al., 2025), helping them develop a stronger sense of belonging and intercultural competence. Addressing these issues across contexts enables the design of holistic approaches that recognize the intertwined nature of cultural diversity and adolescents’ sleep health.

## Conclusions

Considering the importance of sleep health and increasing exposure to intercultural interactions during adolescence, a deeper investigation of the reciprocal associations between these two aspects was needed during this developmental phase. Especially considering that nowadays adolescents’ sleep is a public health concern in many countries, yet there is limited evidence on how adolescents’ social experiences in contemporary societies, characterized by increasing cultural diversity, are intertwined with sleep health. The present study addressed these gaps in the existing literature by testing the longitudinal associations between adolescents’ subjective and objective sleep and the quality of their intercultural interactions in school and during leisure time. Overall, a complex pattern of associations between sleep health and intercultural interaction quality emerged at both the within- and between-person levels, confirming the interplay between adolescents’ well-being and their adaptation to multicultural environments. Negative interactions were consistently associated with lower subjective sleep health, lower sleep efficiency, and shorter sleep duration. Conversely, positive intercultural interactions were positively associated with better sleep efficiency, although these effects were mainly observed at the between-person level.

To conclude, this study extended previous research focused primarily on the complex social environments in which people are embedded, which significantly affect their health. These findings should encourage researchers to take a new look at adolescents' sleep health, considering social aspects typical of multicultural societies (e.g., intercultural interactions) as factors that can influence well-being during adolescence. Furthermore, beyond the theoretical implications, practitioners can better support adolescents from diverse backgrounds in achieving healthier developmental outcomes by recognizing how social and cultural dynamics impact sleep and overall mental health.

## Funding

This work was supported by a grant from the European Research Council (ERC) under the European Union's Horizon 2020 research and innovation programme (ERC—CoG IDENTITIES Grant agreement N. [101002163]; Principal investigator: Elisabetta Crocetti).

## Ethical approval

All procedures performed in this study involving human participants were in accordance with the ethical standards of our institute and with the 1964 Helsinki declaration and its later amendments or comparable ethical standards.

## Informed consent

Active consent from parents was obtained prior to their children’s participation. Active consent was obtained from adolescents of age (18 and older), while underaged adolescents provided their assent to participate in the project.

## Availability statement

Data, analyses, codes, and outputs are publicly available on Open Science Framework at the following link: https://osf.io/fh8sm.

## CRediT authorship contribution statement

**Maria Pagano:** Conceptualization, Formal analysis, Writing – review & editing, Data curation. **Valeria Bacaro:** Conceptualization, Formal analysis, Writing – review & editing, Data curation. **Elisabetta Crocetti:** Conceptualization, Writing – review & editing, Data curation.

## Declaration of competing interest

The authors declare that they have no known competing financial interests or personal relationships that could have appeared to influence the work reported in this paper.

## Appendix Supplementary materials

Image, application 1
