# Supplementary material for: Sleeping in multicultural societies: The longitudinal interplay between adolescents’ sleep health and intercultural interactions
Source: Int J Clin Health Psychol. 2026 Apr 9;26(1):100680. doi: 10.1016/j.ijchp.2026.100680 (PMC13091125; doi:10.1016/j.ijchp.2026.100680)
Supplement: Supplementary file 1 [file mmc1.docx]

**SUPPLEMENTARY INFORMATION FOR:**

**Sleeping in Multicultural Societies: The Longitudinal Interplay Between Adolescents’ Sleep Health and Intercultural Interactions**

**Table S1:** Normality Indices of Variables

**Table S2:** Bivariate Correlations among T1 Study Variables

**Table S3:** Bivariate Correlations among T2 Study Variables

**Table S4:** Bivariate Correlations among T3 Study Variables

**Table S5:** Bivariate Correlations among T4 Study Variables

**Table S6**: Longitudinal Measurement Invariance Tests for Each Study Measure

**Table S7:** Fit indices and Model Comparisons for Random Intercept Cross-Lagged Panel Models

**Table S8:** Sample Participation Across Measurement Waves

**Table S1**

*Normality Indices of Variables*

|  | **T1** | | **T2** | | **T3** | | **T4** | |
| --- | --- | --- | --- | --- | --- | --- | --- | --- |
|  | ***Skewness*** | ***Kurtosis*** | ***Skewness*** | ***Kurtosis*** | ***Skewness*** | ***Kurtosis*** | ***Skewness*** | ***Kurtosis*** |
| **Sleep Health** |  |  |  |  |  |  |  |  |
| Subjective sleep problems | 0.65 | 0.09 | 0.65 | -0.12 | 0.66 | -0.12 | 0.65 | 0.11 |
| Objective sleep efficiency | -1.43 | 4.07 | -1.11 | 1.49 | -1.48 | 2.70 | -1.22 | 1.59 |
| Objective sleep duration | -0.37 | 0.21 | -0.51 | 0.53 | -0.52 | 0.58 | -0.33 | 0.22 |
| **Intercultural interactions at school** |  |  |  |  |  |  |  |  |
| Positive | -0.79 | 1.34 | -1.02 | 2.57 | -0.60 | 1.01 | -0.37 | 0.22 |
| Negative | 1.42 | 2.72 | 1.27 | 1.56 | 1.11 | 1.02 | 0.95 | 0.85 |
| **Intercultural interactions during leisure time** |  |  |  |  |  |  |  |  |
| Positive | -0.64 | 0.96 | -0.45 | 0.47 | -0.57 | 0.87 | 0.03 | 0.11 |
| Negative | 1.30 | 2.21 | 0.99 | 0.48 | 0.93 | 0.64 | 0.55 | 0.11 |

*Note. In a perfectly normal distribution, skewness and kurtosis are equal to zero. However, according to Field (2013), values of skewness and kurtosis between −2 and +2 are typically considered acceptable, particularly for large samples. Other authors suggest more conservative thresholds (e.g., between −1 and +1; Tabachnick & Fidell, 2013). Therefore, values falling within these intervals can be interpreted as indicating approximately normal distributions, whereas values exceeding these limits may reflect substantial deviations from normality.*

**Table S2**

Bivariate Correlations among T1 Study Variables

|  | 1. | 2. | 3. | 4. | 5. | 6. | 7. |
| --- | --- | --- | --- | --- | --- | --- | --- |
| 1. Subjective sleep problems | 1 |  |  |  |  |  |  |
| 2. Objective sleep efficiency | -.05 | 1 |  |  |  |  |  |
| 3. Objective sleep duration | -.11^**^ | .26^***^ | 1 |  |  |  |  |
| 4. Positive intergroup interactions at school | -.03 | .10^*^ | .06 | 1 |  |  |  |
| 5. Negative intergroup interactions at school | .14^***^ | -.04 | -.05 | -.53^***^ | 1 |  |  |
| 6. Positive intergroup interactions during leisure time | -.07 | .08 | .08 | .50^***^ | -.38^****^ | 1 |  |
| 7. Negative intergroup interactions during leisure time | .12^**^ | -.05 | -.07 | -.41^***^ | .53^***^ | -.54^***^ | 1 |

*Note.* ^*^ *p* < .05, ^**^ *p* < .01, ^***^ *p* < .001

**Table S3**

Bivariate Correlations among T2 Study Variables

|  | 1. | 2. | 3. | 4. | 5. | 6. | 7. |
| --- | --- | --- | --- | --- | --- | --- | --- |
| 1. Subjective sleep problems | 1 |  |  |  |  |  |  |
| 2. Objective sleep efficiency | -.09^*^ | 1 |  |  |  |  |  |
| 3. Objective sleep duration | -.09^**^ | .29^***^ | 1 |  |  |  |  |
| 4. Positive intergroup interactions at school | -.02 | .13^**^ | .03 | 1 |  |  |  |
| 5. Negative intergroup interactions at school | .18^***^ | -.06 | -.11^**^ | -.51^***^ | 1 |  |  |
| 6. Positive intergroup interactions during leisure time | -.06 | .17^***^ | .11^**^ | .66^***^ | -.47^***^ | 1 |  |
| 7. Negative intergroup interactions during leisure time | .18^***^ | -.03 | -.10* | -.44^***^ | .70^***^ | -.54^***^ | 1 |

*Note.* ^*^ *p* < .05, ^**^ *p* < .01, ^***^ *p* < .001

**Table S4**

Bivariate Correlations among T3 Study Variables

|  | 1. | 2. | 3. | 4. | 5. | 6. | 7. |
| --- | --- | --- | --- | --- | --- | --- | --- |
| 1. Subjective sleep problems | 1 |  |  |  |  |  |  |
| 2. Objective sleep efficiency | -.04 | 1 |  |  |  |  |  |
| 3. Objective sleep duration | -.08 | .29^***^ | 1 |  |  |  |  |
| 4. Positive intergroup interactions at school | -.09^*^ | .03 | .11^*^ | 1 |  |  |  |
| 5. Negative intergroup interactions at school | .14^***^ | .04 | -.09 | -.56^***^ | 1 |  |  |
| 6. Positive intergroup interactions during leisure time | -.04 | .04 | .012 | .59^***^ | -.41^***^ | 1 |  |
| 7. Negative intergroup interactions during leisure time | .17^***^ | -.02 | -.03 | -.44^***^ | .59^***^ | -.55^***^ | 1 |

*Note.* ^*^ *p* < .05, ^**^ *p* < .01, ^***^ *p* < .001

**Table S5**

Bivariate Correlations among T4 Study Variables

|  | 1. | 2. | 3. | 4. | 5. | 6. | 7. |
| --- | --- | --- | --- | --- | --- | --- | --- |
| 1. Subjective sleep problems | 1 |  |  |  |  |  |  |
| 2. Objective sleep efficiency | -.11^**^ | 1 |  |  |  |  |  |
| 3. Objective sleep duration | -.07 | .29^***^ | 1 |  |  |  |  |
| 4. Positive intergroup interactions at school | -.09^*^ | .07 | .03 | 1 |  |  |  |
| 5. Negative intergroup interactions at school | .20^***^ | -.13^**^ | -.08 | -.58^***^ | 1 |  |  |
| 6. Positive intergroup interactions during leisure time | -.09 | .09 | -.04 | .68^***^ | -.48^****^ | 1 |  |
| 7. Negative intergroup interactions during leisure time | .16^***^ | -.13^*^ | -.07 | -.59^***^ | .66^***^ | -.63^***^ | 1 |

*Note.* ^*^ *p* < .05, ^**^ *p* < .01, ^***^ *p* < .001

**Table S6**

Longitudinal Measurement Invariance Tests for Each Study Measure

|  | **Model fit indices** | | | | | **Model comparison** | | | | | |
| --- | --- | --- | --- | --- | --- | --- | --- | --- | --- | --- | --- |
|  | *χ*_SB_^2^ | *df* | CFI | SRMR | RMSEA [90% CI] | Models | Δχ_SB_^2^ | Δ*df* | *p* | ΔCFI | ΔRMSEA |
| **Subjective sleep problems** | | | | | | | | | | | |
| M1. Configural model | 254. 155 | 134 | .981 | .031 | .025 [.020, .030] |  |  |  |  |  |  |
| M2. Metric model | 281.336 | 146 | .978 | .036 | .025 [.021, .030] | M2-M1 | 27.391 | 12 | .007 | -.003 | 0 |
| **Intergroup interactions at school** | | | | | |  |  |  |  |  |  |
| M1. Configural model | 974.780 | 652 | .980 | .035 | .019 [.017, .022] |  |  |  |  |  |  |
| M2. Metric model | 992.631 | 676 | .980 | .037 | .019 [.016, .021] | M2-M1 | 16.956 | 24 | .851 | 0 | 0 |
| **Intergroup interactions during leisure time** | | | | | |  |  |  |  |  |  |
| M1. Configural model | 991.902 | 652 | .972 | .040 | .022 [.019, .025] |  |  |  |  |  |  |
| M2. Metric model | 1037.958 | 676 | .970 | .045 | .022 [.019, .025] | M2-M1 | 47.624 | 24 | .003 | -.002 | 0 |

*Note.* χ_SB_^2^ = Satorra-Bentler scaled chi-square; *df* = degrees of freedom; CFI = Comparative Fit Index; SRMR = Standardized Root Mean Square Residual; RMSEA [90% CI] = Root Mean Square Error of Approximation and 90% Confidence Interval; Δ = Change in the parameter.

**Table S7**

Fit indices and Model Comparisons for Random Intercept Cross-Lagged Panel Models.

|  | **Model fit indices** | | | | | **Model comparison** | | | | | |
| --- | --- | --- | --- | --- | --- | --- | --- | --- | --- | --- | --- |
| Model | *χ*_SB_^2^ | *df* | CFI | SRMR | RMSEA [90% CI] | Models | Δχ_SB_^2^ | Δ*df* | *p* | ΔCFI | ΔRMSEA |
| **Subjective sleep problems and intercultural interactions at school** | | | | | |  |  |  |  |  |  |
| M1. Baseline | 22.837 | 21 | .999 | .020 | .008 [.000, .025] |  |  |  |  |  |  |
| M2. Time invariant cross lagged paths | 43.667 | 33 | .996 | .033 | .016 [.000, .027] | M2-M1 | 20.160 | 12 | .064 | -.003 | .005 |
| M3. M2 with T2-T4 time invariant within time correlations | 43.228 | 39 | .998 | .035 | .009 [.000, .022] | M3-M2 | 1.893 | 6 | .929 | .002 | -.007 |
| M4. M3 with autoregressive paths fixed | 53.683 | 45 | .997 | .028 | .012 [.000, .023] | M4-M3 | 10.194 | 6 | .117 | -.001 | .001 |
| M5. M4 with constrained covariates | 92.451 | 72 | .992 | .033 | .015 [.000, .023] | M5-M4 | 40.165 | 27 | .049 | -.005 | .003 |
| **Objective sleep (efficiency and duration) and intercultural interactions at school** | | | | | | | | | | | |
| M1. Baseline | 43.453 | 38 | .998 | .044 | .011 [.000, .023] |  |  |  |  |  |  |
| M2. Time invariant cross lagged paths | 73.101 | 62 | .997 | .056 | .012 [.000, .022] | M2-M1 | 29.562 | 24 | .200 | -.001 | .001 |
| M3. M2 with T2-T4 time invariant within time correlations | 87.819 | 74 | .996 | .075 | .012 [.000, .021] | M3-M2 | 14.686 | 12 | .259 | -.001 | .000 |
| M4. M3 with autoregressive paths fixed | 103.909 | 82 | .994 | .095 | .015 [.000, .022] | M4-M3 | 14.443 | 8 | .071 | -.002 | .003 |
| M5. M4 with constrained covariates | 146.669 | 118 | .992 | .114 | .014 [.004, .021] | M5-M4 | 42.510 | 36 | .211 | -.002 | -.001 |
| **Subjective sleep problems and intercultural interactions during leisure time** | | | | | | | | | | | |
| M1. Baseline | 24.929 | 21 | .998 | .021 | .012 [.000, .027] |  |  |  |  |  |  |
| M3. M2 with T2-T4 time invariant within time correlations | 40.259 | 33 | .997 | .027 | .013 [.000, .025] | M2-M1 | 15.271 | 12 | .227 | -.001 | .001 |
| M4. M3 with autoregressive paths fixed | 42.951 | 39 | .998 | .028 | .009 [.000, .022] | M3-M2 | 3.243 | 6 | .778 | .001 | -.004 |
| M4. M3 with autoregressive paths fixed | 46.070 | 45 | 1 | .031 | .004 [.000, .019] | M4-M3 | 3.596 | 6 | .731 | .002 | -.005 |
| M5. M4 with constrained covariates | 79.194 | 72 | .997 | .037 | .009 [.000, .019] | M5-M4 | 33.749 | 27 | .173 | -.003 | .005 |
| **Objective sleep (efficiency and duration) and intercultural interactions during leisure time** | | | | | |  |  |  |  |  |  |
| M1. Baseline | 66.014 | 38 | .991 | .048 | .025 [.014, .034] |  |  |  |  |  |  |
| M2. Time invariant cross lagged paths | 83.028 | 62 | .993 | .056 | .017 [.004, .026] | M2-M1 | 18.993 | 24 | .752 | .002 | -.008 |
| M3. M2 with T2-T4 time invariant within time correlations | 104.433 | 74 | .990 | .078 | .018 [.009, .026] | M3-M2 | 21.217 | 12 | .047 | -.003 | .001 |
| M4. M3 with autoregressive paths fixed | 113.194 | 82 | .990 | .101 | .018 [.009, .025] | M4-M3 | 9.392 | 8 | .310 | .000 | .000 |
| M5. M4 with constrained covariates | 152.636 | 118 | .988 | .118 | .016 [.007, .022] | M5-M4 | 38.816 | 36 | .344 | -.002 | -.002 |

*Note.* χ_SB_^2^ = Satorra-Bentler scaled chi-square; *df* = degrees of freedom; CFI = Comparative Fit Index; SRMR = Standardized Root Mean Square Residual; RMSEA [90% CI] = Root Mean Square Error of Approximation and 90% Confidence Interval; Δ = Change in the parameter.

**References**

Tabachnick, B. G., & Fidell, L. S. (2013). *Using multivariate statistics, 6th ed Boston*. Ma: Pearson.

Field, A. (2024). *Discovering statistics using IBM SPSS statistics*. Sage publications limited.
